# Treatment of fecal incontinence—is there a light in the end of the tunnel?

**DOI:** 10.1007/s00508-024-02369-7

**Published:** 2024-05-07

**Authors:** Stefan Riss, Christopher Dawoud

**Affiliations:** Department of General Surgery, Division of Visceral Surgery, Medical University Vienna, Währinger Gürtel 18–20, 1090 Vienna, Austria

**Keywords:** Fecal incontinence, Sacral neuromodulation, Sphinkeeper®, Artificial bowel sphincter, Anal band, Minimally invasive surgery, SIMPLYFI

## Abstract

Fecal incontinence (FI) is a common disease with higher incidence rates in the elderly population. Treatment of affected patients remains challenging and ranges from conservative management to surgical techniques. Despite all efforts patients often undergo several therapeutic measurements to achieve reasonable functional improvements.

Although sacral neuromodulation still remains a key therapy with success rates up to 80%, a significant number of patients do not respond sufficiently and require further treatment.

Several artificial bowel sphincter devices exist, which can lead to better functional control in selected patients. Notably, complications after these surgeries do occur frequently and the need for implant replacement is still considerable high.

A novel anal band, developed by Agency for Medical Innovations (A.M.I., Austria) is currently under evaluation. This device, composed of silicone and polyester, is placed around the anus outside the external sphincter muscle complex aiming to improve stool continence via mechanical pressure. Early results of this new operation are eagerly awaited.

## Short report

Fecal incontinence is a devastating disease with a high physical and mental burden for those who are affected [1]. It is still considered a taboo topic; thus, exact prevalence rates are difficult to obtain. However, current literature suggests an overall prevalence of 8.3%, with increasing numbers in elderly patients [2].

The etiology of FI is multifactorial and several causes often contribute to the development of the involuntary control of feces [2]. Notably, in order to choose an appropriate treatment a careful diagnostic work up is essential.

Conservative management, including dietary changes, medication for stool regulation, pelvic floor exercises, should be offered primarily and can be beneficial in patients with milder forms of FI. Noteworthy, a higher number of patients continue to suffer from the inability to control stool and require further and more invasive therapies.

Unfortunately, available operations are limited and healing rates are still moderate. Sacral neuromodulation can be regarded as a key treatment modality with success rates reaching up to 80% in patients with FI [3]. Due to the fact, that not all patients respond adequately to electric stimulation, a test phase is required before definite implantation [4]. The Sphinkeeper®, the successor of the Gatekeeper®, represents another technique to treat patients with primarily passive FI [5]. Although, up to ten implants are placed in a circular fashion into the intersphincteric groove, only around half of the patients benefit from this type of therapy [6].

Artificial bowel sphincter has also been implanted with good functional success rates, but were associated with considerable complications rates and the frequent need of surgical removal [7].

**Fig. 1:**
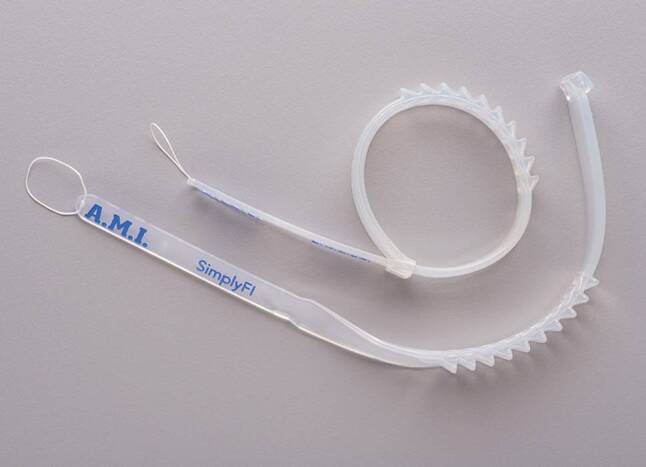
Shows two of the new silicone anal bands “SimplyFI” from A.M.I

We are currently conducting an approval study of a novel anal band, produced by Agency for Medical Innovations (A.M.I., Austria) (see Fig. 1). This small, elastic band consists of biocompatible material (silicone and polyester) and is placed around the anus outside the external sphincter muscle complex. The implantation is performed through two 2‑centimeter incisions at 3‑ and 9‑o’clock lithotomy position. The aim of this innovative technique is to achieve a better functional control by increased mechanical pressure. At the same time, defecation should still be possible, without the necessity of deflating a balloon as it was required in previous implants [8]. The use of a minimally invasive implantation technique and the use of a new material should also reduce the infection rates and subsequently the number of device replacements. Together with A.M.I., J.M. Devesa has been involved in developing the new medical product, which was also based on a previous experience using a Flat Drain Type Jakson-Pratt® for the purpose of anal encirclement [9]. In that study, a considerable number of patients required an explant of the device, which was mainly necessary due to the breakdown of the product itself. Consequently, it might be possible that the new silicon band overcomes these limitations and offers a significant better outcome.

If this simple and new technique is a potential candidate to improve the burden of patients with FI too is still unclear. First results are eagerly awaited and can possibly expected to be available in summer 2024.
